# Evidence for Replacement of an Infected Synthetic by a Biological Mesh in Abdominal Wall Hernia Repair

**DOI:** 10.3389/fsurg.2015.00067

**Published:** 2016-01-08

**Authors:** Agneta Montgomery, Friedrich Kallinowski, Ferdinand Köckerling

**Affiliations:** ^1^Department of Surgery, Skane University Hospital, Malmö, Sweden; ^2^Department of Surgery, Asklepios Hospital Harburg, Hamburg, Germany; ^3^Department of Surgery, Centre for Minimally Invasive Surgery, Vivantes Hospital Berlin, Academic Teaching Hospital of Charité Medical School, Berlin, Germany

**Keywords:** hernia, mesh infection, biological mesh, mesh replacement, mesh complication

## Abstract

**Introduction:**

The incidence of deep infection using a synthetic mesh in inguinal hernia repair is low and reported to be well below 1%. This is in contrast to incisional hernia surgery where the reported incidence is 3% respective 13% comparing laparoscopic to open mesh repair reported in a Cochrane review. Main risk factors were long operation time, surgical site contamination, and early wound complications. An infected mesh can be preserved using conservative treatment were negative pressure wound therapy (VAC^®^) could play an important role. If strategy fails, the mesh needs to be removed. This review aims to look at evidence for situations were a biological mesh would work as a replacement of a removed infected synthetic mesh.

**Materials and methods:**

A literature search of the Medline database was performed using the PubMed search engine. Twenty publications were found relevant for this review.

**Results:**

For studies reviewed three options are presented: removal of the infected synthetic mesh alone, replacement with either a new synthetic or a new biological mesh. Operations were all performed at specialist centers. Removal of the mesh alone was an option limited to inguinal hernias. In ventral/incisional hernias, the use of a biological mesh for replacement resulted in a very high recurrence rate, if bridging was required. Either a synthetic or a biological mesh seems to work as a replacement when fascial closure can be achieved. Evidence is though very low.

**Conclusion:**

When required, either a synthetic or a biological mesh seems to work as a replacement for an infected synthetic mesh if the defect can be closed. It is, however, not recommended to use a biological mesh for bridging. Mesh replacement surgery is demanding and is recommended to be performed in a specialist center.

## Introduction

Reduced recurrence rates can be achieved by using standardized surgical techniques for mesh reinforcement in hernia surgery (1–3). Accordingly, several different types of meshes are used worldwide in hernia surgery. In the US alone, some 800,000 inguinal hernia (4) and 400,000 ventral hernia, including primary and incisional (5), operations are performed annually. In Sweden, 16,000 inguinal and 7,000 ventral hernias are reported on an annual basis in the national registers. In Germany, 275,000 inguinal and 100,000 ventral hernia operations are carried out annually.

In a review (6), the reported incidence of mesh-related infections following hernia repair was between 1 and 8% in different series. The incidence was influenced by the underlying comorbidities, type of mesh, surgical technique, and the strategy used to prevent infections. Risk factors to determine the onset of mesh infection were a prolonged operation time (7, 8), the extent of contamination of the surgical site (9), and early complications of the wound (seroma, hematoma, and infection) (8). In the Cochrane review of laparoscopic versus open surgical techniques for ventral or incisional hernia repairs, the overall infection rate was 13% after open and 3% after laparoscopic mesh repair (10).

Prevention of mesh infections continues to be the best strategy (11). Not all infections necessitate mesh removal. In the Cochrane review, only 3.3% of meshes had to be removed following open and 0.7% following laparoscopic ventral and incisional hernia repairs (10). It was possible to preserve 17 (55%) of meshes through conservative treatment in a case series of 31 infected meshes after incisional hernias repair (7). In a study on in ventral hernia repairs by Liang et al., a total of 30 out of 407 (7.4%) were re-operated due to an infection and the mesh could be saved in 10 out of these 30 (33%) (9). Reoperations were performed evenly spread from operation up to 10 years after the primary operation. In another series, it was possible to preserve 12 out of 13 (92%) were VAC^®^ was used in addition in 11 patients (12). The rate of mesh removal due to infection following inguinal hernia repairs was reported to be 0.13% (13). The interval between hernia operation and mesh removal could be up to 10 years or longer.

In a review by Darehzereshki et al. including eight retrospective studies, with a total of 1,229 patients comparing different biological to synthetic mesh repair in ventral and incisional hernias. It was demonstrated that biological grafts were associated with significantly fewer wound infections (*p* < 0.00001) but with no difference in recurrence rate (14).

The aim to look at evidence for situations were a biological mesh would work as a replacement of a removed infected synthetic mesh.

## Materials and Methods

A literature search of the Medline database was performed using the PubMed search engine. The following key words were used: biological mesh, replacement of infected mesh, ventral hernia A*N*D infected mesh, inguinal hernia A*N*D infected mesh, mesh infection A*N*D biological mesh, infected synthetic mesh A*N*D biological mesh. Two thousand five hundred one citations were found. After checking the title and abstracts, 20 publications remained included in this study. Seven of these publications, four case series and three case reports, do report on the replacement of a synthetic by a biological mesh (Table 1).

**Table 1:** **Characteristics and outcomes of studies reporting on replacement of infected synthetic meshes with either a synthetic or biologic mesh in ventral/incisional hernia repair**.

Reference	Study design	Patients (*n*)	Mesh for replacement	Intervention details	Follow-up time	Outcome
Birolini et al. (15)	Retrospective case series	41	HW PP	Single stage	74 months	27 uneventful
				Single surgeon		10 (24%) inf
				Onlay		1 mesh removal
						3 recur
						1 EC fistula
Albino et al. (5)	Retrospective cases series	27	PADM	Two stages	32 months	6 wound rupt
				6 bridging		5 inf
						5 (19%) recur (all bridged rep)
Rosen et al. (16)	Retrospective case series	128 in total	102 Strattice	Single stage	22 months	61 (48%) inf
		45 (35%) inf*	16 Alloderm	87 rr mesh		28 major
			5 Biodesign	40 ip mesh		33 minor
			4 Xenmatrix	70% comp sep		40 (31%) recur
			4 BioA	6% bridging		
Guerra (17)	Retrospective case series	13	PADM^®^	Single stage	22 months	1 inf
				2 bridged		1 seroma
						2 recur (both bridged repairs)
Cavallaro et al. (18)	Case report	2	Bovine pericardium graft	Single stage	5 years	0 inf
				rr		0 recur
Peppas et al. (19)	Case report	1 EC fistula	Porcine tissue	Two stages	6 months	0 inf
		Two meshes	Collamend^®^			0 recur
		PTFE and PP				
Coccolini et al. (20)	Case report	2	Collamend^®^ Surgisis	Single stage	36 months	0 inf 0 recur

*HW = heavy weight, PP = polypropylene, PTFE = polytetrafluoreten, rr = retro rectus, ip = intra peritoneal, PADM = porcine acellular dermal matrix, mo = months, inf = infection, recur = recurrence, rupt = rupture, EC = enterocutanous, rep = repair*.

*Mesh infections cannot be identified for individual meshes.

The treatment options were removal of the infected mesh alone, replacement of the infected with a new synthetic mesh, and replacement of an infected synthetic with a biological mesh.

## Results

### Removal of the Infected Mesh Alone

In the following two studies, a total of 47 patients with mesh infection were treated by means of partial or complete mesh removal. Neither a synthetic nor a biological mesh was implanted to replace the explanted mesh.

In a retrospective case series by Akyol et al., 15 mesh removals were performed after inguinal hernia repair because of chronic mesh infection in 14 males and 1 female with a median age of 52 years (range 35–75 years) (13). At the time of presentation, 13 patients had chronic sinus at explanation, while 2 had abscesses. The interval from hernia repair to mesh removal was 4–204 months. The infected meshes were completely removed. None of the patients had the transversalis fascia reinforced, due to thickening by fibrosis from the former mesh. Follow-up was performed median 62 months (range 16–115 months). Infection resolved successfully in all patients. One patient reported paresthesia and another developed a recurrent hernia.

In a retrospective case series by Tolino et al., 32 mesh removals were performed due to chronic infection, 22 after incisional and 10 after inguinal hernia repair (8). The interval from repair to mesh removal ranged from 4 to 60 months. A total of 51 operations in the 32 patients were needed for definitive treatment, including partial or total mesh removal. The average follow-up was 40 months (range 30–97). Five hernia recurrences and one intestinal fistula were observed after incisional mesh removal. One recurrence and one fistula developed after inguinal hernia mesh removal.

### Replacement of the Infected with a New Synthetic Mesh

In a single surgeon case series by Birolini et al., a 16-year retrospective review based on a prospective protocol was carried out in 41 patients having had ventral hernias surgery with their meshes removed (15). A total of 27 patients had a supportive infection and 14 had an exposed mesh. Bowel resection or an associated contaminated procedure was performed in 15 patients. An onlay polypropylene mesh was used for replacement in all patients. In the short-term follow-up, all, except one mesh, could be preserved. Three recurrences were seen after a mean follow-up of 74 months, out of which one was associated with an intestinal fistula. A total of 95% of the patients were considered cured from their chronic mesh infection. It was concluded that onlay polypropylene mesh yielded favorable outcomes, for high-risk ventral hernia patients, having an infected synthetic mesh removed in a single-stage repair setting.

### Replacement of the Infected with a Biological Mesh

In the following six studies (three case series and three case reports), a total of 92 patients with mesh infection were treated by mesh removal followed by implant of a biological mesh in ventral hernia patients.

In a retrospective case series, Albino et al. reported on 27 patients with an infected synthetic mesh treated with a multi-staged approach (5). The initial surgical procedure consisted of abdominal exploration with debridement and mesh removal followed by VAC^®^ therapy. In the second stage, all patients underwent component separation and hernia repair reinforced by porcine acellular dermal matrix (PADM). Primary fascial closure was achieved in 21 (78%) of patients (19 meshes placed underlay and 2 onlay). Bridging was performed in six (22%) patients. The average follow-up was 32 months (range 8–52 months). Six (22%) patients were found to have wound dehiscence and five (19%) of these had had clinical evidence of a surgical site infection. Wound healing was achieved in all patients in average after 8 weeks (2–60 weeks). Five (19%) patients developed a recurrent hernia. Both bridging and a postoperative infection were found to increase the risk of a hernia recurrence (*p* = 0.03 and 0.001, respectively).

In a single institution, Rosen et al. reported on 128 patients who had a single-stage reconstruction using a biological mesh in a contaminated field, of whom 45 (35%) were operated on for a simultaneous removal of a contaminated mesh (16). The mesh removal patients were not reported on separately. A total of 27% of operations were considered “dirty” according to the CDC classification and would probably include most of the mesh infected patients. A total of 66% had a retromuscular and 31% an intraperitoneal mesh repair. Component separation was performed in 70% of patients and fascial closure was achieved in 94%. Overall wound morbidity was seen in 61 patients (48%), of whom 28 were re-operated and 33 managed by local treatment of the infection. All wounds resolved within 60 days. At a mean follow-up of 22 months, 31% recurrences were seen. It can be concluded that using a biological mesh in these situations is safe, but the long-term durability seems to be less favorable, even when fascial closure has been achieved.

In a retrospective case review by Guerra, 13 patients had an infected synthetic mesh removed after former incisional hernia surgery (17). Mesh replacement was performed with a porcine-derived acellular dermal matrix. The mean age was 60 years. Comorbidity was high. Facial closure was achieved in 11 and bridging in 2 patients. One wound infection, one seroma, and two hernia recurrences (both bridged patients) were observed at a median follow-up of 22 months. It was concluded that outcomes were favorable in high-risk patients with infected synthetic mesh if bridging was avoided.

Two patients were presented in a case report by Cavallaro et al. where one preperitoneal Prolene^®^ mesh and one retromuscular polypropylene mesh were extracted and replaced with a retromuscular bovine pericardium graft. No complication and no recurrences were reported after 5 and 4 years, respectively. Closure of the gap was not reported on (18).

In a case report, Peppas et al. described drainage of an infected ePTFE together with a macro porous onlay polypropylene mesh for 1 month. The meshes were extracted and replaced by porcine onlay mesh (19). No complications were reported up to 6 months.

Two patients were reported by Coccolini et al. having a surgical site infection after a double-layered PP-e PTFE retromuscular mesh repair (20). The first patient had a surgical site infection that discovered with substantial abdominal wall tissue loss 2 weeks after the operation for a recurrence. After 2 years of conservative treatment, the patient underwent mesh removal and retromuscular reconstruction using an acellular porcine dermal collagen cross-linked implant (CollaMend™). The second patient had an infection resulting in a sinus. The mesh was removed and replaced by a porcine mucosal non-cross-linked implant (Surgisis™). At 37 respective 35 months after the operation, the patients demonstrated no evidence of recurrence. The description of the technique used in these two patients implicates a bridging procedure (20).

## Discussion

Mesh procedures are standard practice for surgical repair of both inguinal, ventral and incisional hernias (1–3). As the number of hernias treated worldwide continues to grow, also the number of hernia meshes implanted each year rise inexorably. Mesh infection rate in inguinal hernia surgery is below 1% and is not regarded as a clinical problem. However, surgeons often have to deal with mesh infections after ventral and incisional hernia surgery, which is estimated to be between 1 and 8% (6). The primary treatment modality is conservative. This is successful in eliminating mesh infection in over 50% of cases without mesh removal (7, 10). The most common bacterial agent is *Staphylococcus aureus*. With increasing proportion of methicillin resistance (MRSA), the treatment options in long-standing wound infections might be problematic to handle (11). There are no recommendations on how long a conservative regime is acceptable. Polypropylene and polyester meshes can be saved in a higher proportion that a laminar mesh-like ePTFE. Extensive ePTFE mesh infections are best managed by mesh explantation (11). Pros and cons must though be weighed against each other according to the scenario presented. If conservative treatment fails, the mesh must be explanted (8, 15).

In mesh infection, biological meshes are increasingly used for replacement as synthetic meshes by some are regarded as contraindicated (5). The publications included in the present review demonstrated that there were three approaches that could be taken depending on the individual patient situation. The first option was to remove the infected mesh without a new implant. This is the most common option after inguinal hernia surgery, since the transversalis fascia is thickened by fibroses after the mesh removal (13). Using this approach, no inguinal hernia recurrence was seen on mean follow-up of 62 months in a case series (13). This does, however, not apply for incisional and ventral hernias. Tolino et al. reported on a recurrence rate of 23% after removal of an infected mesh following incisional hernia operation without reimplantation of a new mesh (8).

The second option was to replace the infected polypropylene mesh with a new polypropylene mesh (15). The short-term results showed a relative uneventful postoperative course after mesh replacement in 27 patients. Six (22%) patients developed a minor wound infection and were treated with dressings and antibiotics, five (19%) patients had wound infections requiring debridement and one required complete mesh removal. On follow-up, there were three hernia recurrences, one with an enterocutaneous fistula. Ninety-five percent of the patients undergoing mesh replacement were considered cured from chronic mesh infection after a mean follow-up of 74 months (15).

**Figure 1:**
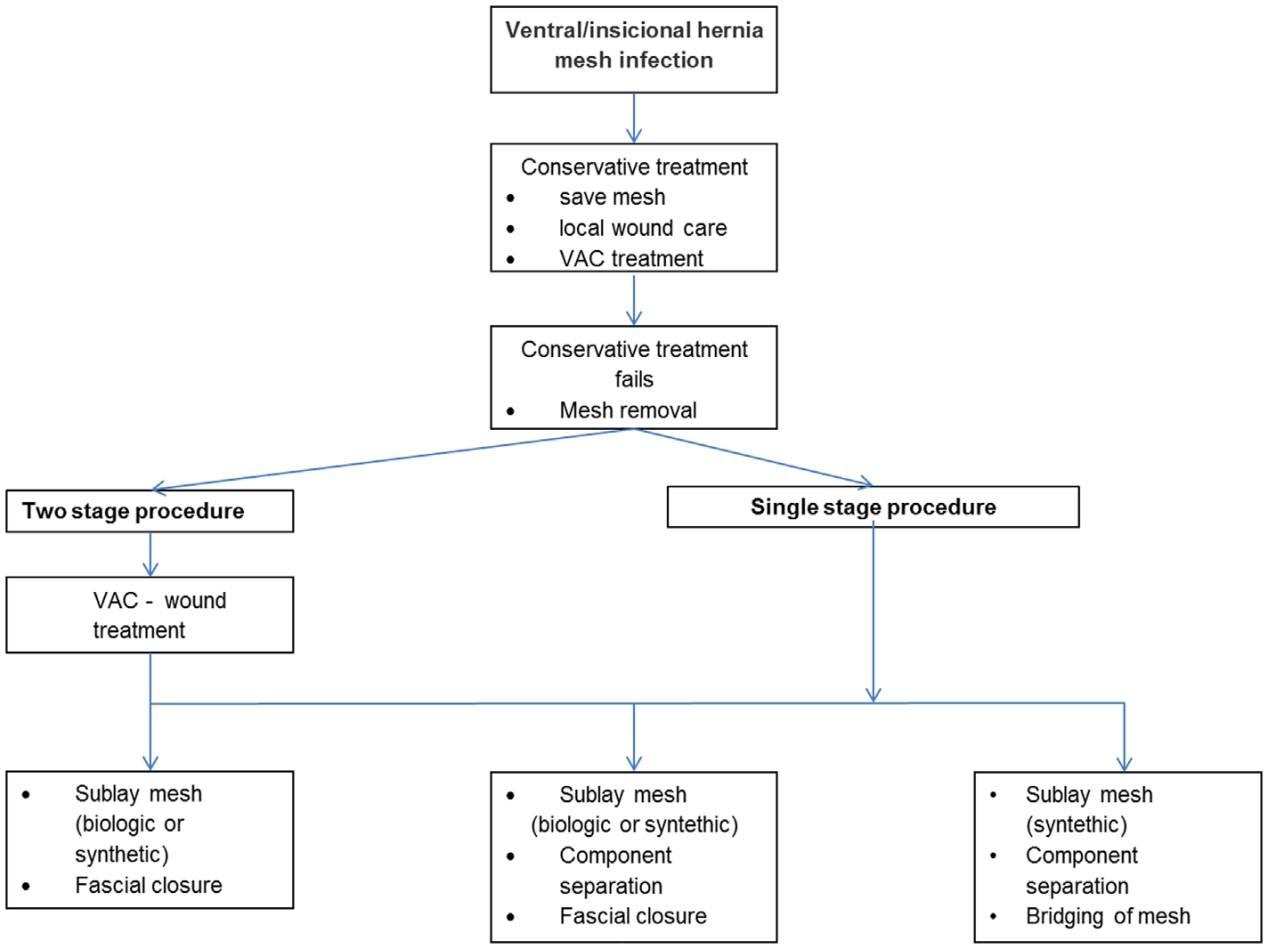
**Algorithm for treatment of ventral/incisional hernia mesh infection**.

The third option was to replace the explanted synthetic mesh with a biological mesh (5, 16–20). Long-term results were successful only if bridging was omitted (5, 16, 17). An unacceptably high recurrence rate was observed following bridging with biological meshes (5, 16, 17). When bridging was avoided, good results were obtained for replacement of an infected synthetic mesh with a biological (5, 16, 17). An algorithm for treatment of ventral/incisional hernia mesh infection is presented in Figure 1.

It can be concluded that a mesh can be saved in more than half of patients suffering from an infection after implantation of a synthetic mesh for an incisional hernia. If mesh explanation is necessitated a replacement seems safe either using a synthetic or a biological mesh if fascia could be closed. Bridging seems to result in a high failure rate using a biological mesh. Further studies are needed to create a better evidence-based platform for specific therapeutic decision-making.

## Author Contributions

AM: literature search, selection of the literature, review of the literature, and writing of the manuscript. F Ka: literature search, selection of the literature, review of the literature, and revision of the manuscript. F Kö: literature search, selection of the literature, review of the literature, and revision of the manuscript.

## Conflict of Interest Statement

The authors declare that the research was conducted in the absence of any commercial or financial relationships that could be construed as a potential conflict of interest.

## Appendix

### BioMesh Study Group

Ferdinand Köckerling (Chairman), Stavros Antoniou, René Fortelny, Frank A. Granderath, Markus Heiss, Franz Mayer, Marc Miserez, Agneta Montgomery, Salvador Morales-Conde, Filip Muysoms, Alexander Petter-Puchner, Rudolph Pointner, Neil Smart, Marciej Smietanski, and Bernd Stechemesser.

### Aim

The BioMesh Study Group has set itself the task of identifying how best to use biological meshes for the various indications. The first step toward achieving that goal is to compile systematic reviews of the different indications on the basis of the existing literature. The available literature sources will be evaluated in accordance with the Oxford Centre for Evidence-Based Medicine-Levels of Evidence (March 2009). Next, based on the review findings, corresponding Statements and Recommendations are to be formulated in a Consensus Conference for the use of biological meshes for the different indications. The findings of the Consensus Conference are then to be summarized for a joint publication. This present publication is a part of the project undertaken by the BioMesh Study Group.

## References

[1] Scott NW McCormack K Graham P Go PM Ross SJ Grant AM. Open mesh versus non-mesh for repair of femoral and inguinal hernia. Cochrane Database Syst Rev (2002) 4:CD002197. 10.1002/14651858.CD002197 12519568 PMC13574228

[2] McCormack K Scott NW Go PM Ross S Grant AM EU Hernia Trialists Colloboration. Laparoscopic techniques versus open techniques for inguinal hernia repair. Cochrane Database Syst Rev (2003) 1:CD001785. 10.1002/14651858.CD001785 12535413 PMC8407507

[3] Luijendijk RW Hop WC van den Tol MP de Lange DC Braaksma MM Jzermans JN A comparison of suture repair with mesh repair for incisional hernia. N Engl J Med (2000) 343(6):392–8. 10.1056/NEJM200008103430603 10933738

[4] Rutkow IM. Demographic and socioeconomic aspects of hernia repair in the United States in 2003. Surg Clin North Am (2003) 83(5):1045–51. 10.1016/S0039-6109(03)00132-4 14533902

[5] Albino FP Patel KM Nahabedian Y Attinger CE Bhanot P. Immediate, multistaged approach to infected synthetic mesh: outcomes after abdominal wall reconstruction with porcine acellular dermal matrix. Ann Plast Surg (2015) 75(6):629–33. 10.1097/SAP.0000000000000186 24667884

[6] Falagas ME Kasiakou SK Mesh-related infections after hernia repair surgery. Clin Microbiol Infect (2005) 11:3–8. 10.1111/j.1469-0691.2004.01014.x 15649297

[7] Stremitzer S Bachleitner-Hofmann T Gradl B Gruenbeck M Bachleitner-Hofmann B Mittelboeck M Mesh graft infection following abdominal hernia repair: risk factor evaluation and strategies of mesh graft preservation. A retrospective analysis of 476 operations. World J Surg (2010) 34(7):1702–9. 10.1007/s00268-010-0543-z 20372901

[8] Tolino MJ Tripoloni DE Ratto R Garcia MI. Infections associated with prosthetic repairs of abdominal wall hernias: pathology, management and results. Hernia (2009) 13:631–7. 10.1007/s10029-009-0541-y 19657591

[9] Liang MK Nguyen MT Berger RL Hicks SC Kao LS. Abdominal reoperation and mesh explantation following open ventral hernia repair with mesh. Am J Surg (2014) 208(4):670–6. 10.1016/j.amjsurg.2013.10.024 25241955

[10] Sauerland S Walgenbach M Habermalz B Seiler CM Miserez M. Laparoscopic versus open surgical techniques for ventral or incisional hernia repair. Cochrane Database Syst Rev (2011) 16(3):CD007781. 10.1002/14651858.CD007781.pub2 21412910 PMC13389912

[11] Collage RD Rosengart MR. Abdominal wall infections with in situ mesh. Surg Infect (Larchmt) (2010) 11(3):311–8. 10.1089/sur.2010.029 20583867

[12] Meagher H Clarke Moloney M Grace PA. Conservative management of mesh-site infection in hernia repair surgery: a case series. Hernia (2015) 19(2):231–7. 10.1007/s10029-013-1069-8 23504138

[13] Akyol C Kocaay F Orozakunov E Genc V Bayram IK Cakmak A Outcome of the patients with chronic mesh infection following open inguinal hernia repair. J Korean Surg Soc (2013) 84:287–91. 10.4174/jkss.2013.84.5.287 23646314 PMC3641368

[14] Darehzereshki A Goldfarb M Zehetner J Moazzez A Lipham JC Mason RJ Biologic versus nonbiologic mesh in ventral hernia repair: a systematic review and meta-analysis. World J Surg (2014) 38(1):40–50. 10.1007/s00268-013-2232-1 24101015

[15] Birolini C de Miranda JS Utiyama EM Rasslan S. A retrospective review and observations over a 16-year clinical experience on the surgical treatment of chronic mesh infection. What about replacing a synthetic mesh on the infected surgical field? Hernia (2015) 19:239–46. 10.1007/s10029-014-1225-9 24509890

[16] Rosen JM Krpata DM Ermlich B Blatnik JA. A 5-year clinical experience with single-staged repairs of infected and contaminated abdominal wall defects utilizing biologic mesh. Ann Surg (2013) 257:991–6. 10.1097/SLA.0b013e3182849871 23426340

[17] Guerra O Noncrosslinked procine-derived acellular dermal matrix for single-stage complex abdominal wall herniorrhaphy after removal of infected synthetic mesh: a retrospective review. Am Surg (2014) 80(50):489–95. 24887729

[18] Cavallaro A Lo Menzo E Di Vita M Zanghi A Cavallaro V Veroux PF Use of biological meshes for abdominal wall reconstruction in highly contaminated fields. World J Gastroenterol (2010) 16(15):1928–33. 10.3748/wjg.v16.i15.1928 20397274 PMC2856837

[19] Peppas G Makris MC Falagas ME Biological mesh for abdominal wall hernia synthetic mesh multidrug-resistant *Pseudomonas aeruginosa* infection: report of case. Surg Today (2011) 41:717–20. 10.1007/s00595-010-4326-9 21533949

[20] Coccolini F Catena F Ansaloni L Neri F Gazzotti F Lazzareschi D An innovative abdominal wall repair technique for infected prosthesis: the Eskimo technique. Ulus Travma Acil Cerrahi Derg (2011) 17(4):354–8. 10.5505/tjtes.2011.56767 21935836

